# Branched-chain amino acid metabolism supports Roseobacteraceae positive interactions in marine biofilms

**DOI:** 10.1128/aem.02411-24

**Published:** 2025-02-11

**Authors:** Han Cui, Shuaitao Wang, Shen Fan, Hongan Long, Jinshui Lin, Wei Ding, Weipeng Zhang

**Affiliations:** 1 MOE Key Laboratory of Evolution & Marine Biodiversity and Institute of Evolution & Marine Biodiversity, Ocean University of China 535359 , Qingdao, China; 2 MOE Key Laboratory of Marine Genetics and Breeding and College of Marine Life Sciences, Ocean University of China 506915 , Qingdao, China; 3 College of Life Sciences, Yan'an University 105849 , Yan'an, China; University of Delaware, Lewes, Delaware, USA

**Keywords:** marine biofilm, Roseobacteraceae, interspecies interaction, branched-chain amino acids

## Abstract

Interspecies interactions are key factors affecting the stability of microbial communities. However, microbial interactions in marine biofilms, which constitute up to 80% of the microbial biomass in certain marine environments, are not well understood. We addressed this knowledge gap by coculturing four marine biofilm-derived Roseobacteraceae strains (*Leisingera aquaemixtae* M597, *Roseibium aggregatum* S1616, *Alloyangia pacifica* T6124, and *Sulfitobacter indolifex* W002) in 14 single carbon sources. Overall, 140 coculture experiments revealed 39.3% positive interactions compared to 8.3% negative interactions. When the carbon source was consumed by only one strain, the interaction between the strains was more likely to be positive. The interaction between S1616 and M597, when cultured in D-gluconic acid, was further studied as an example. S1616–M597 coculture displayed a higher D-gluconic acid consumption rate than S1616 monoculture, whereas M597 could not use D-gluconic acid as the sole carbon source. The supernatant of S1616 monoculture supported the growth of M597, and branched-chain amino acids in the supernatant were consumed. Transcriptomic analysis suggested that M597 induced the expression of genes for branched-chain amino acid biosynthesis in S1616. Additionally, metagenomic analysis revealed the wide distribution and a strongly correlated co-occurrence of the four strains in global oceanic biofilms. Together, our findings show that interspecies positive interactions are prevalent among marine-biofilm Roseobacteraceae, and the interactions are likely to be mediated by branched-chain amino acids metabolism.

**IMPORTANCE:**

Interspecies interactions are crucial for microbial community structure and function. Despite well-studied social behaviors in model microorganisms, species interactions in natural marine biofilms especially Roseobacteraceae with complex metabolic pathways are not well understood. Our findings suggest that positive microbial interactions, which can be mediated by branched-chain amino acid biosynthesis, are common among marine-biofilm Roseobacteraceae. This study provides new insights into microbial interactions and the ecology of marine biofilms.

## INTRODUCTION

Interspecies interactions are prominent determinants in the structure and functions of microbial communities (1, 2). Based on their impact on the interacting partners, these interactions can be classified into three categories: positive, negative, or neutral. Positive interactions occur when both species benefit from the interactions, or when one species benefits without impacting the growth of the other (3). Negative interactions occur when one species inhibits the growth or activity of another one (4–6). Lastly, when one species has little or no influence on the other species, interactions are classified as neutral.

Previous studies (7, 8) have revealed positive interactions between microbes living in certain environments. Interactions among 20 soil bacteria were investigated, and the common positive interactions among culturable bacteria were determined (7). A study of two human pathogens cooccurring in an oral cavity suggested that dual-species biofilms accumulate higher biomass and cell numbers than monoculture biofilms (8). Whereas contrasting evidence suggested that microbes rarely cooperate with each other (9, 10). A study of interactions among 72 bacterial species isolated from permanent rainwater pools in a beech tree forest revealed that, out of 180 cocultures, only two showed cooperative relationships (9). The pairwise interactions observed between 12 microbial species isolated from the guts of mice living in a well-defined, nutrient-rich environment supported the idea that negative interactions dominate between microbial species (10).

Mechanistically, positive interactions can be mediated in diverse ways. Methanotrophic archaea can degrade methane to produce compounds that serve as electron donors for sulfate-reducing bacteria, aiding these bacteria in overcoming energy limitations (11). Two different bacterial genotypes can survive on growth-inhibiting media through amino acid exchange (12). In addition, metabolic byproducts by a community member, such as oligomers of N-acetylglucosamine, ammonia, organic acids, acetate, and pyruvate, sustain the survival of other species within the community that are unable to utilize polysaccharides directly (13). In contrast, negative interactions may involve bacteria secreting bacteriocins and antimicrobial peptides to eliminate competing species, thereby establishing a competitive advantage (6).

Compared to studies of bacteria on land, fewer studies have focused on interspecies interactions among marine microbes. Marine biofilms are comprised of diverse microbes attached to biotic or abiotic surfaces, such as coastal stone surfaces, microplastics, and animal body surfaces (14, 15). Based on recent studies, marine biofilms constitute up to 80% of the biomass in certain habitats (16) and have very high microbial diversities with a large number of novel species (17, 18). Over 60% of the functional genes in microbiomes of marine biofilms are still unannotated (18). The microbes within marine biofilms are expected to interact with each other because active signal transduction genes and the respective signal molecules are abundant in marine biofilm communities (19, 20). Many bacteria in marine biofilms are auxotrophic and they rely on other heterotrophic community members to produce certain metabolites (e.g., vitamins) (21), also implying interspecies interaction.

Bacteria of the Roseobacteraceae family (previously known as the *Roseobacter* group) are prominent members of marine biomes. Through genomic and molecular studies of Roseobacteraceae bacteria isolated from marine biofilms, we previously demonstrated their novel roles in thiosulfate oxidation, which may contribute to biofilm formation and thus the stability of the community (22). Analysis of global ocean metagenomic data suggests that Roseobacteraceae bacteria are specifically abundant in biofilms in various environments across different ocean provinces (22). Biofilm formation stabilizes metabolism in Roseobacteraceae bacteria when grown under high-temperature conditions (23), suggesting that this group of bacteria has adapted to the biofilm-associated lifestyle. Moreover, Roseobacteraceae respond quickly to take advantage of carbon source availability (24), suggesting that environmental carbon sources significantly affect bacterial interactions within a marine biofilm. Thus, employing Roseobacteraceae to study microbial interactions in marine biofilms is of ecological importance.

In the present study, to explore microbial interactions in marine biofilms, four Roseobacteraceae strains were studied through coculture experiments with various carbon sources. A consortium of two selected strains was further studied using transcriptomics, gene mutation, and physiological experiments to understand the metabolic mechanisms determining the interaction.

## RESULTS

### Positive interactions between four Roseobacteraceae strains

In a project of marine biofilm bacterial isolation (25), 843 strains were isolated from stone surface biofilms immersed in a subtidal zone of Qingdao, China, using agar plate-based isolation. In the current study, four Roseobacteraceae strains, *Leisingera aquaemixtae* M597, *Roseibium aggregatum* S1616, *Alloyangia pacifica* T6124, and *Sulfitobacter indolifex* W002 (genomic information displayed in Fig. S1; Table S1) were used to study interspecies interactions. M597, S1616, and W002 had only one chromosome, with sizes of 3.8 Mb, 5.8 Mb, and 3.4 Mb, respectively (Fig. S1). T6124 possessed two chromosomes, the sizes of which were 1.2 Mb and 2.6 Mb (Fig. S1). M597, S1616, T6124, and W002 had 4,130, 6,170, 4,972, and 3,611 open reading frames (ORFs), respectively, of which 2,393, 3,264, 2,815, and 2,724 could be annotated by searching against the Kyoto Encyclopedia of Genes and Genomes (KEGG) database (Table S1). Other information, including the numbers of rRNA genes, tRNA genes, ORFs, and KEGG-annotated ORFs, has been also provided in Table S1. To infer the relationships between the strains included in the present study and previously reported Roseobacteraceae strains, a phylogenetic tree was built using 31 essential marker genes extracted from the genomes of our strains and 80 reference strains downloaded from the National Center for Biotechnology Information (NCBI) (Fig. S2). The four strains were located in distinct branches (Fig. S2), suggesting that they were a representative mix of Roseobacteraceae species.

**Fig 1:**
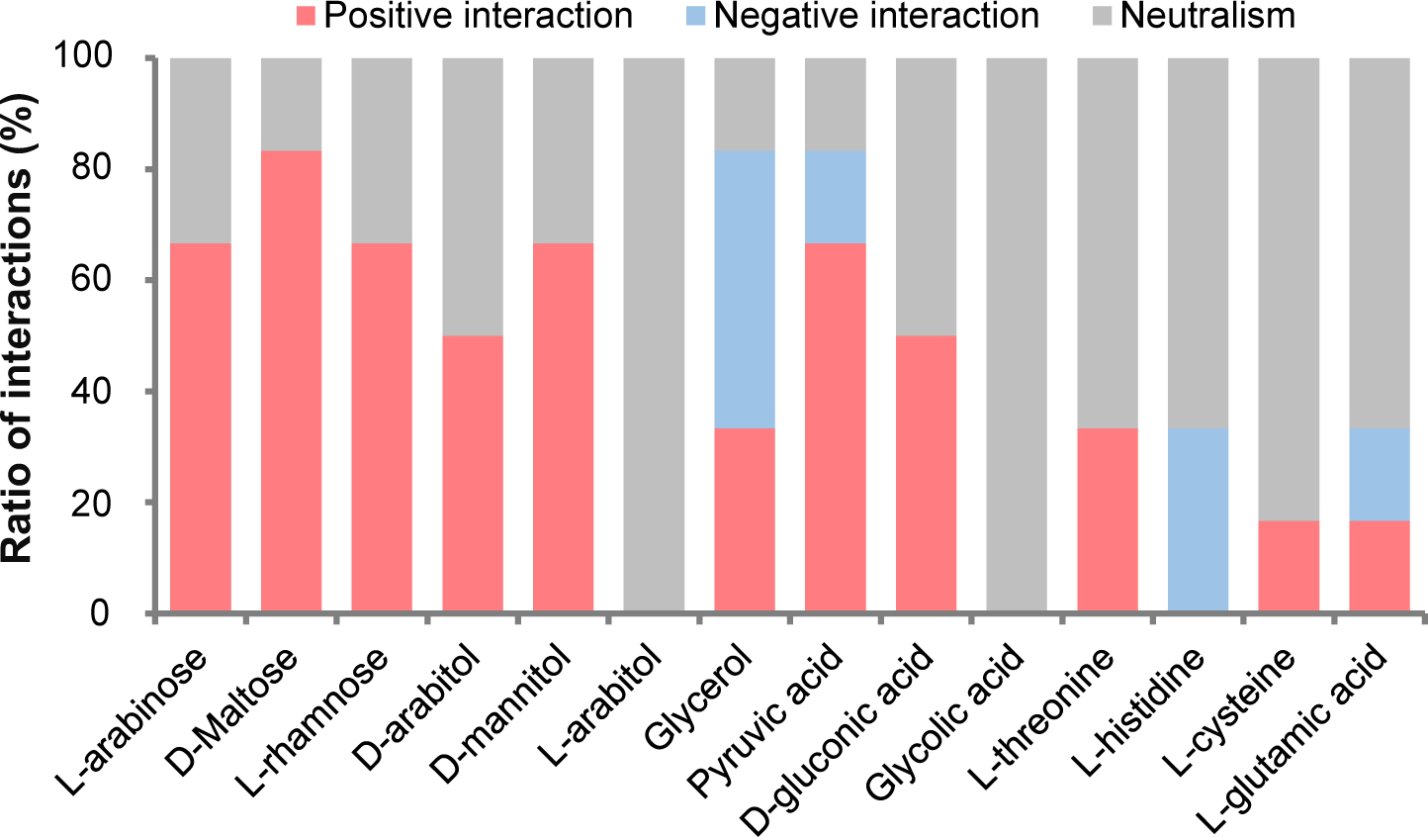
Interactions among the four strains during coculture experiments with 14 sole carbon sources. Number of microbial interactions among all cocultures grown on different carbon sources is shown. The experiments were conducted with three biological replicates.

We next investigated the pairwise interactions among the four strains by coculturing or monoculturing them with different single carbon sources. A total of 14 single carbon sources spanning different carbon categories, including saccharides, organic acids, amino acids, and alcohols were used: D-arabinose, D-arabitol, D-gluconic acid, D-maltose, D-mannitol, glycerol, glycolic acid, L-arabitol, L-cysteine, L-glutamic acid, L-histidine, L-rhamnose, L-threonine, and pyruvic acid. Many of these carbon sources are common in marine environments. For example, glycolic acid is released by marine phytoplankton (26). D-glucuronic acid is the major sugar composition of marine sponges (27). Based on the three biological replicates of each group, we calculated the distribution pattern of the data using the Shapiro-Wilk test and found that each group had good repeatability and belonged to a normal distribution. Students’ *t*-test of the cell density was performed to detect positive and negative interactions between the bacterial strains, with *P*-values corrected using the false discovery rate (FDR) method. A positive interaction was determined when a coculture attained significantly (*P*-value < 0.05) higher cell density than the corresponding monocultures. For example, during monoculture, M597 did not utilize L-arabinose, L-rhamnose, D-arabinitol, or D-mannitol, but when M597 was cocultured with S1616, a substantial increase in cell density was observed (Fig. S3). Whereas a negative interaction was defined when the cell density of the coculture was significantly (*P*-value < 0.05) lower than that of at least one of the corresponding monocultures. For most carbon sources and pairs of strains, positive microbial interactions (39.3%, *n* = 33) were more prevalent than negative interactions (8.3%, *n* = 7) (Fig. S3). Among the carbon sources, except for glycolic acid, L-histidine, and L-arabitol, all the other 11 carbon sources included positive interactions (Fig. 1).

Results of the coculture experiments were also visualized in a scatter plot (Fig. S4) based on the ratio between coculture and monoculture. AB_co_average_/Max_mono_average_ > 1 (AB_co_average_ is the cell density average of three biological replicates of the coculture, while Max_mono_average_ is the cell density average of three biological replicates of the larger value of species A or B in monocultures) indicated positive interactions, while AB_co_average_/Max_mono_average_ < 1 indicated negative interactions. Thus, the result further showed the prevalence of positive interactions. In addition, it seemed that the type of interaction occurring between a pair of strains was partially dependent on the carbon source. If the carbon source is consumed by only one strain, the interaction between the strains tends to be positive. To support this notion, we further classified the positive and negative interactions into two cases: (i) both strains can use the carbon source; (ii) only one strain can use the carbon source. As a result, the first case only occurred in 5 of the 33 positive interactions, while the second case occurred in the remaining 28 positive interactions (Fig. S6). By contrast, the ratio of second case/first case was much smaller among the negative interactions (4/3) than among the positive interactions (28/5) (Fig. S5). Together, the coculture experiments showed positive interactions commonly occurred among the studied microbes.

### Validation of the interaction between S1616 and M597

*R. aggregatum* S1616 and *L. aquaemixtae* M597, cocultured in the presence of D-gluconic acid as the single carbon source, were selected to further explore the mechanisms of positive interaction. We confirmed the positive interaction between S1616 and M597 in coculture by counting colony-forming units (CFUs). The CFU number of S1616–M597 coculture was 1.3 × 10^9^, while S1616 monoculture reached only 9 × 10^7^ CFUs and no CFU was observed for M597 monoculture (Fig. S6). Moreover, to confirm the stable coexistence of these two strains, 16S rRNA gene sequencing was performed on coculture samples collected for 14 days at 2-day intervals. Details of the 16S amplicon sequences are in Table S2. During the initial 6 days, the relative abundance of S1616 was higher than that of M597, and it increased continuously (Fig. S7). Afterward, the abundance of S1616 began to decrease and stabilized at around 50% (Fig. S7). This cyclic fluctuation between both S1616 and M597 suggested cross-feeding. In addition, the consumption of D-gluconic acid in the coculture and monocultures was examined through biochemical assays, which showed that D-gluconic acid consumption in the coculture was higher than that in the monocultures (Fig. S8). These results suggested that the two strains formed a stable consortium to promote carbon source consumption.

### Mechanistic study of the positive interactions between S1616 and M597

**Fig 2:**
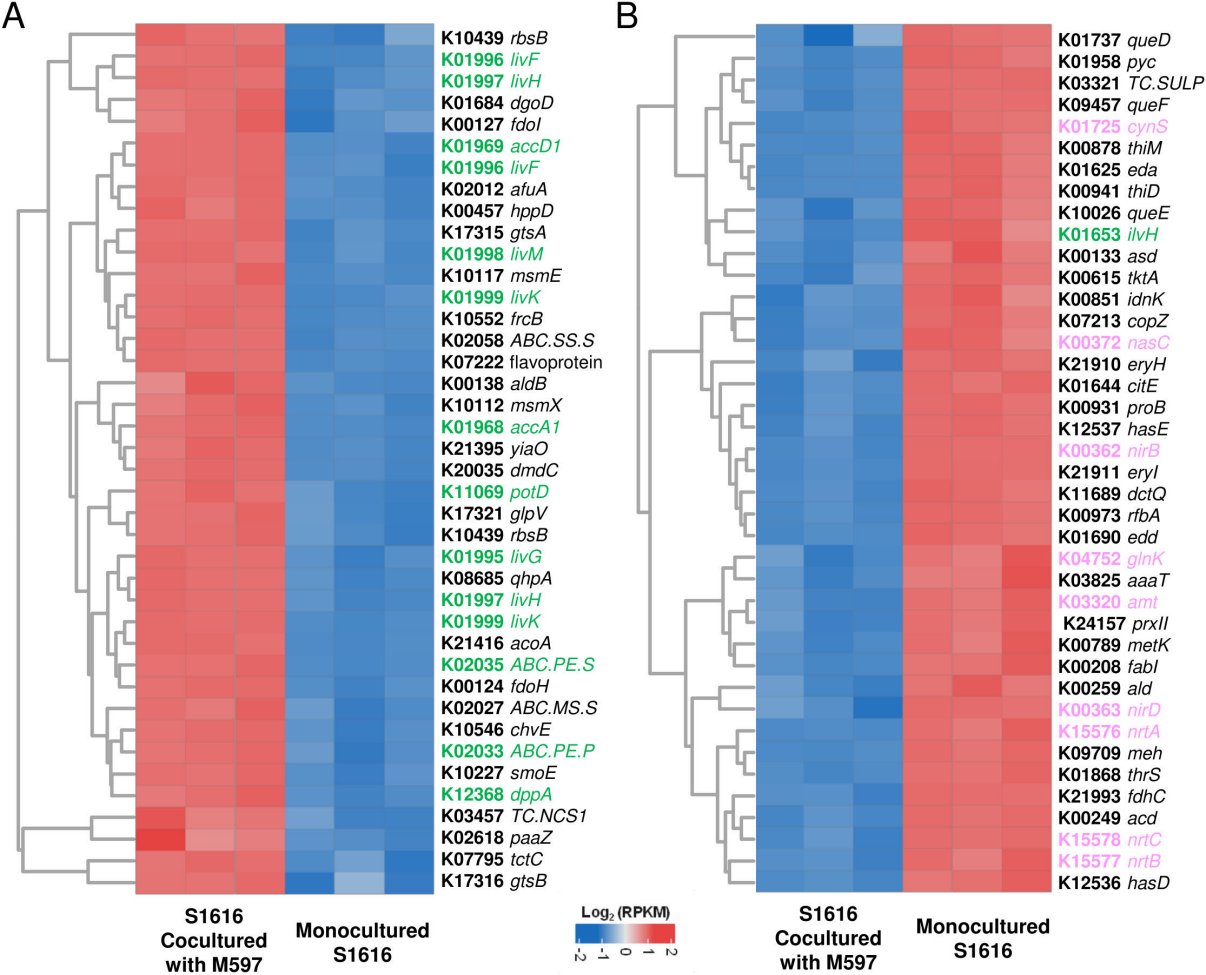
Top 40 upregulated (**A**) and downregulated (**B**) genes in S1616 when cocultured with M597. Genes involved in amino acid metabolism are colored green and those involved in nitrogen metabolism are colored pink. Statistical analysis based on Reads Per Kilobase Million values was performed using a two-sided Student’s *t*-test with a threshold of fold change > 4 and *P*-value < 0.05. *P*-values were corrected with the FDR method. Functional description of selected key genes: *livF*, branched-chain amino acid transport system ATP-binding protein; *livH*; branched-chain amino acid transport system permease protein; *livK*, branched-chain amino acid transport system substrate-binding protein; *accA1,* 3-methylcrotonyl-CoA carboxylase alpha subunit; *accD1,* 3-methylcrotonyl-CoA carboxylase beta subunit; *ilvH*, acetolactate synthase I/III small subunit; *glnK,* nitrogen regulatory protein P-II 2; *nirB*, nitrite reductase large subunit; *nirD*, nitrite reductase small subunit; *nrtC*, nitrate/nitrite transport system ATP-binding protein.

The above results indicated that S1616 was able to transform D-gluconic acid into certain metabolites that were secreted into the extracellular space and consumed by M597. To identify the underlying metabolic pathways, the transcriptomes of the S1616–M597 coculture and S1616 monoculture in D-gluconic acid were sequenced and comparatively analyzed. The transcriptome information is summarized in Table S3. The alteration of gene expression in S1616 was statistically evaluated by conducting a two-tailed Students’ *t*-test using the reads per kilobase million (RPKM) values of the coculture versus monoculture and *P*-values were corrected with the FDR method. Among the 6,087 KEGG-annotated genes, 515 genes were found to be significantly (*P*-value < 0.05 and fold change > 4) upregulated in S1616 when cocultured with M597, whereas 251 genes were significantly downregulated (Fig. S9). Many of the upregulated genes in coculture, such as *livK* (K01999, branched-chain amino acid transport system substrate-binding protein), *livF* (K01996, branched-chain amino acid transport system ATP-binding protein), and *livH* (K01997, branched-chain amino acid transport system permease protein), are associated with the transport of branched-chain amino acids. Furthermore, the upregulation of key enzymes involved in L-leucine metabolism (e.g., *accA1*, K01968, 3-methylcrotonyl-CoA carboxylase alpha subunit; and *accD1*, K01969, 3-methylcrotonyl-CoA carboxylase beta subunit) was witnessed in the coculture (Fig. 2A); these genes are involved in the transformation of branched-chain amino acids into acetoacetate (28). In comparison, genes related to nitrate, nitrite, and ammonia metabolism (e.g., *nirD*, K00363, nitrite reductase small subunit; *glnK*, K04752, and nitrogen regulatory protein P-II 2) were downregulated in S1616 when cocultured with M597 (Fig. 2B).

**Fig 3:**
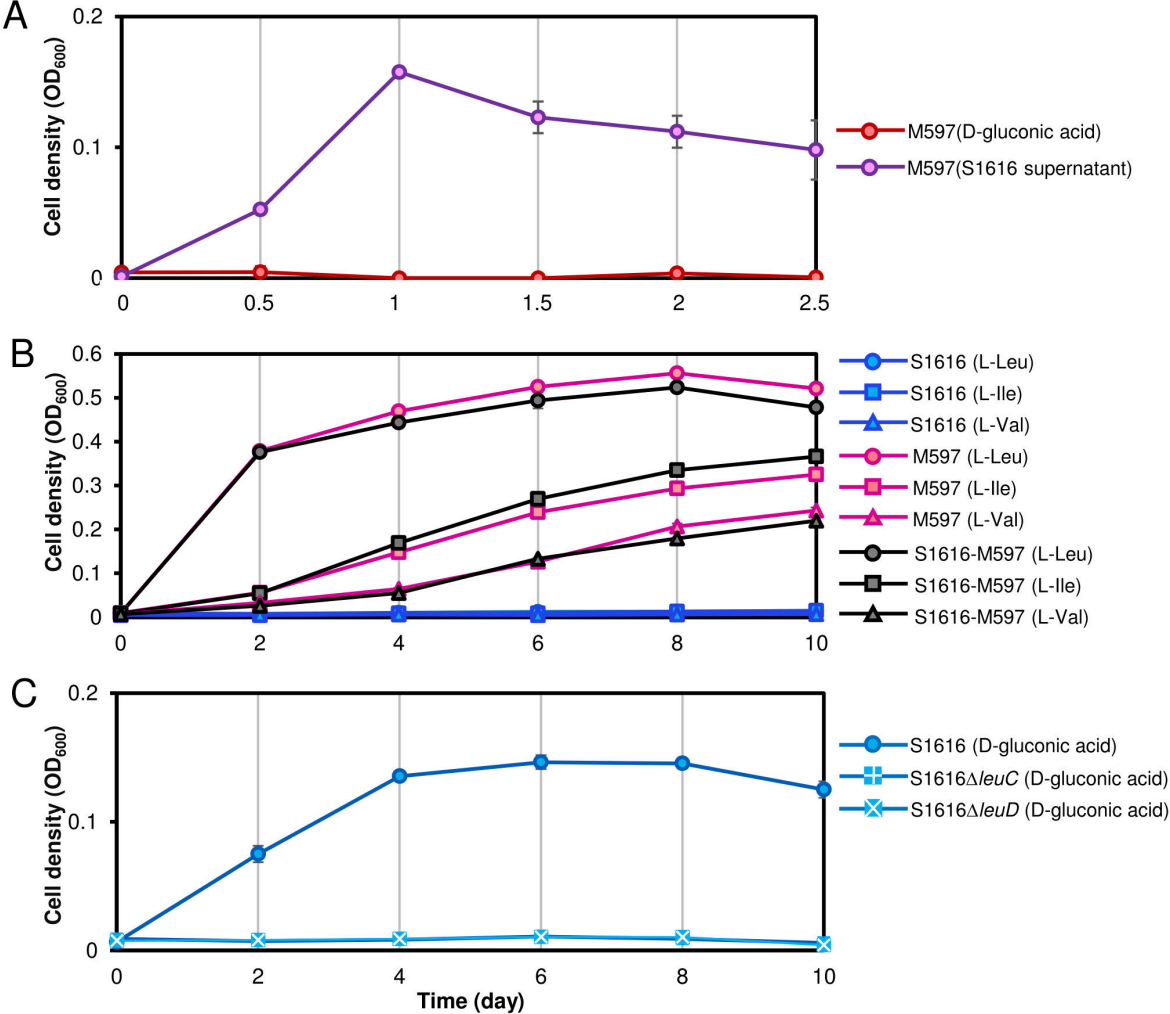
Growth of S1616, M597, and the S1616 mutants. (**A**) Growth of M597 in D-gluconic acid or the supernatant of S1616 metabolites grown in D-gluconic acid. (**B**) S1616 and M597 growth (cocultured or monocultured) in L-leucine, L-isoleucine, and L-valine. (**C**) Growth of wild type and mutant strains of S1616 in D-gluconic acid. *leuC*, 3-isopropylmalate dehydratase large subunit. *leuD*, 3-isopropylmalate dehydratase small subunit. In all figures, dots represent the mean of three biological replicates and error bars indicate standard deviations.

**Fig 4:**
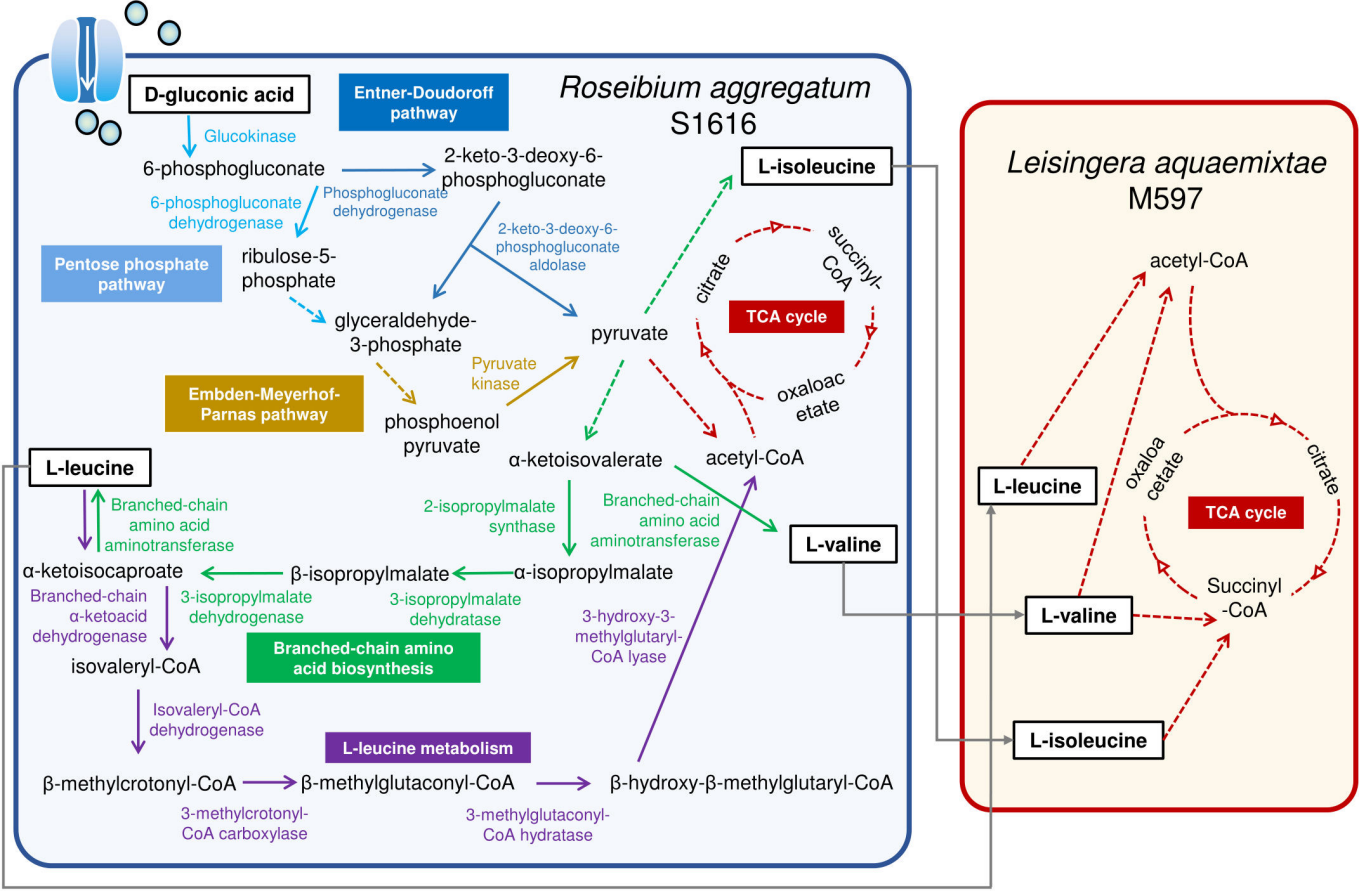
Schematic model showing the metabolic interactions between S1616 and M597. S1616 supports M597 growth by transforming D-gluconic acid into branched-chain amino acids. This figure was drawn based on the results of the genomic and transcriptomic analyses, as well as the growth experiments. Different pathways are shown by arrows of different colors. Solid lines indicate one-step reactions, while dashed lines indicate multi-step reactions.

The upregulation of genes related to branched-chain amino acid transport and metabolism in S1616, after it was cocultured with M597, indicated that more branched-chain amino acids are required for the growth of both strains in coculture. Branched-chain amino acids generated by S1616 are likely to be key molecules in promoting the growth of itself and M597. To test this assumption using experiment, the supernatant of S1616 grown in D-gluconic acid was used to culture M597, which led to substantial growth in biomass after 24 h (Fig. 3A). To explore the role of L-leucine, L-isoleucine, and L-valine in the growth of S1616 and M597, concentrations of the three amino acids in S1616 supernatants, before or after growth of S1616 and M597, were measured. The results revealed the consumption of L-leucine, L-isoleucine, and L-valine by M597 (Fig. S10). To confirm the role of L-leucine, L-isoleucine, and L-valine in the growth of the two strains, the compounds were used as sole carbon sources to culture S1616 and M597. The results revealed that S1616 could not utilize any of these branched-chain amino acids when provided as a single carbon source, while M597 grew very well in the presence of any of the three amino acids (Fig. 3B). Moreover, the S1616-M597 coculture produced a similar biomass as the M597 monoculture (Fig. 3B), suggesting that branched-chain amino acids are likely to be primarily consumed by M597 in the coculture consortium. To verify the role of branched-chain amino acid metabolism in supporting the growth of S1616, key genes involved in L-leucine biosynthesis (i.e., *leuC* encoding 3-isopropylmalate dehydratase large subunit and *leuD* encoding 3-isopropylmalate dehydratase small subunit) were knocked out to obtain the mutant strains S1616*ΔleuC* and S1616*ΔleuD*, respectively. A culture experiment revealed that neither S1616*ΔleuC* nor S1616*ΔleuD* could grow with D-gluconic acid as the sole carbon source (Fig. 3C), suggesting that biosynthesis of branched-chain amino acids is essential for S1616 to grow in D-gluconic acid. Using these results, a schematic model was prepared to illustrate the interactions between S1616 and M597 (Fig. 4). Briefly, D-gluconic acid was consumed by S1616 and then converted to pyruvate through the pentose phosphate pathway and the Embden–Meyerhof–Parnas pathway. Pyruvate was then converted to L-leucine, L-isoleucine, and L-valine, which were used by S1616 for anabolism and secreted into the extracellular space to support the growth of M597 (Fig. 4).

### Distribution and interaction patterns of the four strains in marine biofilms

**Fig 5:**
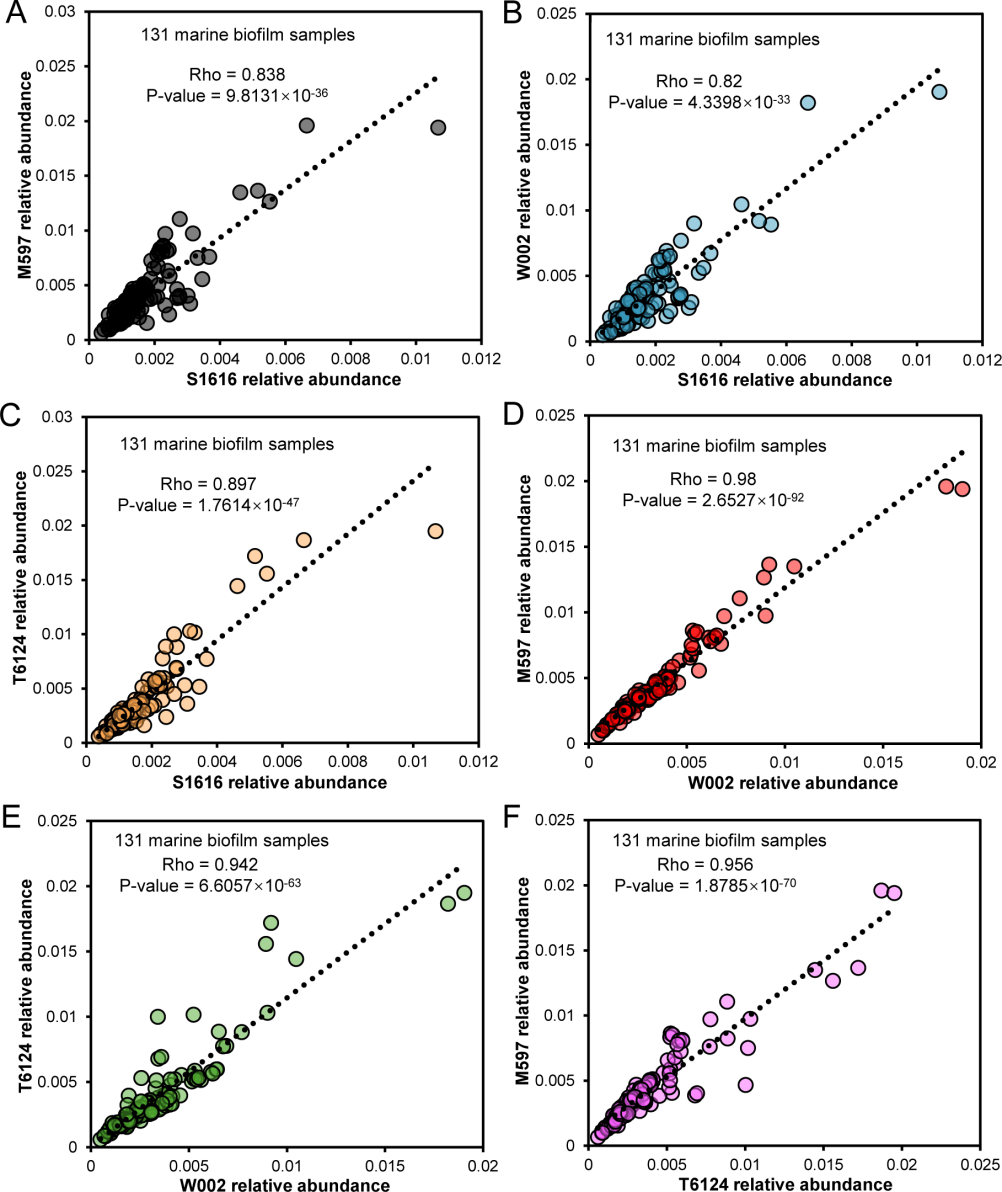
Distribution and correlation of pairwise strains among S1616, M597, W002, and T6124 in 131 marine biofilms. The correlations have been indicated by Spearman’s Rho values and *P*-values. (**A**) S1616–M597. (**B**) S1616–W002. (**C**) S1616–T6124. (**D**) W002–M597. (**E**) W002–T6124. (**F**) T6124–M597.

The distribution patterns of the four strains in natural marine biofilm metagenomes were examined to reveal the ecological significance of the interaction between these strains. A total of 131 biofilms were collected from the Pacific Ocean, the Indian Ocean, and the Atlantic Ocean. Among these biofilms, 18 biofilms were collected from the metal panels (18), 13 from the rock surfaces (18, 22), 76 from the plastic panels (18), and 24 from the surfaces of microplastic or glass particles (15). The detailed information on these biofilms and the corresponding metagenomic data sets have been summarized in Table S4. Based on distribution analysis, S1616, M597, T6124, and W002 exhibited relatively high abundance in the biofilm range of 0.04%–1.07%, 0.07%–1.96%, 0.06%–1.95%, and 0.05%–1.9%, respectively (Fig. 5). Significant coexistences between each pair of strains were detected, with Spearman coefficients ranging from 0. 82 to 0.98 and *P*-values lower than 0.05 (Fig. 5).

## DISCUSSION

In the present study, the coculture experiments support the prevalence of positive interactions among Roseobacteraceae in marine biofilms. In many cases, coculturing with a partner strain enables the utilization of a broader range of carbon sources (Fig. 1). Moreover, the positive interactions can be largely supported by sharing branched-chain amino acids between species.

Positive microbial interactions are often a result of sharing metabolites. For example, polysaccharide-degrading microbes of the marine community secrete extracellular hydrolases to transform complex polysaccharides into simpler products that can be utilized by the other members of the community (29). Methanotrophic archaea, through the degradation of methane and the production of its byproducts, can serve as electron donors for sulfate-reducing bacteria, thereby helping these bacteria overcome energy limitations (11). The results of the present study highlight the role of branched-chain amino acids in mediating microbial interactions. The utilization of branched-chain amino acids as the sole carbon source by M597 (Fig. 3 and 4) suggests that relevant pathways are key determinants in the interaction between the two strains. It was suggested that during monoculture growth, the donor strain re-import some of the metabolites they release, achieving a steady state of production and consumption (30). When another strain emerges in the same growth environment and begins to consume some of the metabolites produced by the donor strain, the donor strain must increase its production rates of the metabolite to maintain its current growth level (30). Consistently, during coculture, the upregulated branched-chain amino acid transport system in S1616 is used to uptake amino acids (Fig. 2). After M597 utilizes the branched-chain amino acids in the coculture environment, S1616 upregulates the expression of its transport system to uptake more branched-chain amino acids, and meanwhile, S1616 will produce more amino acids to meet the demands of both strains. On the other hand, S1616 is likely to benefit in other ways, such as the easier metabolism of certain nitrogen sources, as indicated by the downregulation of genes related to nitrate and nitrite metabolism.

Ecologically, the positive microbial interactions regulated by branched-chain amino acids are of great significance for marine biofilms and microbes. Branched-chain amino acids are molecules closely related to cell growth and are precursors of branched-chain fatty acids, which are the main components of cell membranes and biofilm matrix (31), and thus may play roles in stabilizing the biofilm community. Moreover, branched-chain amino acids can enter the tricarboxylic acid (TCA) cycle, which has been shown to play an important role in the adaptation of marine Roseobacteraceae to low-temperature environments (32). Furthermore, branched-chain amino acids play a pivotal role in modulating bacterial virulence, including the induction of virulence gene expression (33). In addition, branched-chain amino acids can directly activate *Rhodobacter capsulatus* alarmone synthetase/hydrolase, thereby appropriately regulating the cellular stringent response to cope with amino acid deficiency or salt stress in the environment (34). Therefore, effective utilization of branched-chain amino acids through positive interactions may provide a foundational basis for community stability and the response to environmental change.

Finally, metagenomic analysis has revealed the wide distribution and strong correlation of the four strains in global oceanic biofilms (Fig. 5). This finding has further extended the ecological significance of these strains, as well as the relevant interactions, which may contribute to the stability of biofilm communities and even the whole marine biosphere. To conclude, we have shown that branched-chain amino acid biosynthesis is likely to play a role in mediating mutualistic cooperative activities among Roseobacteraceae species in marine biofilms. Considering the oligotrophic conditions of most marine environments, microbes living in the seas probably prefer cooperative relationships, rather than competition, that facilitate the consumption of limited carbon sources. Altogether, our findings provide new insights into microbial interaction and the ecology of marine biofilms.

## MATERIALS AND METHODS

### Bacterial isolation and coculture experiments

The marine biofilms were sampled using sterile cotton tips from the surface of stones immersed in the subtidal zone of Qingdao (120.145° E, 39.915° N), China. The biofilm samples were serially diluted (10, 10^2^, 10^3^, 10^4^, 10^5^, 10^6^, and 10^7^-fold) using marine broth 2216E liquid medium (Becton Dickinson and Company, USA). Aliquots of diluted cultures (100 µL) were evenly spread on the marine 2216E agar plates (Becton Dickinson and Company, USA) and the plates were incubated at 25°C for 1–7 days. Obtained colonies were examined under a dissecting microscope for morphological characterization and conspicuous colonies were picked for subculturing. After subculturing more than five generations on marine 2216E agar plates, the pure colonies were seeded into a 2216E liquid medium for genomic DNA extraction and taxonomic identification. Genomic DNA was extracted using the TIANamp Genomic DNA Kit (Tiangen Biotech, China). PCR was performed to amplify the 16S rRNA genes of the extracted DNA using the universal primers 27F (5′-AGAGTTTGATCCTGGCTCAG-3′) and 1492R (5′-GGTTACCTTGTTACGACTT-3′). The PCR reaction mix (20 µL) contained 1 µL of DNA, 1 µL of primers, 7 µL of sterile water, and 10 µL of Taq DNA Polymerase (Sangon Biotech, China). Sanger sequencing of amplified DNA was performed at the Beijing Genomics Institute, China. The 100 bp of unstable base sequences were removed from the front and back end to get high-quality sequences. Subsequently, taxonomic classification of the 16S rRNA gene sequences was done using the website of NCBI. Redundant strains were identified using a 16S identity cut-off of 99.9%.

Strains grown overnight in Marine Broth 2216E medium were used for coculture experiments. The individually cultured bacterial cells were washed twice using carbon-free media (3,687 × *g*, 5 min) to remove the residual carbon sources. The ratio of cell density between the two strains was 1:1 and the initial cell inoculation biomass for monoculture and coculture was the same with a final cell density of 0.005 (OD_600_). Subsequently, bacterial strains were monocultured and cocultured in pairs using 14 single carbon sources. The carbon source concentration in the media was 0.1%, while the concentrations of other components were the same as in the media used for enrichment culture. All cultures were incubated under static conditions at 25°C, and OD_600_ values of the cultures were recorded on days 1, 3, 5, 7, and 9 to obtain the maximum OD_600_ value. First, we performed the Shapiro-Wilk test on the obtained data using SPSS software (version 28.0.1.1) to assess whether the data followed a normal distribution. Based on the results, with *P*-values > 0.05, we concluded that the experimental data follow a normal distribution. Subsequently, the differences in the cell densities of monocultures and cocultures were examined using a two-tailed Students’ *t*-test. This statistical analysis was performed based on the average value of maximum OD_600_ derived from three biological replicates. For each strain pair and carbon source, the coculture result was compared with the two monocultures one by one. To exclude false positives caused by multiple tests, *P*-values were corrected with FDR using the “fdrtool” package in R software. *P*-value < 0.05 and mean biomass values > 0.05 (based on the error of the instrument we used, the maximum error at 600 nm is approximately 0.003 Abs) for both comparison groups were considered indicative of a significant difference between the two groups in this study.

### Colony-forming unit counting

The coculture of S1616 and M597 on D-gluconic acid and the monoculture of S1616 were diluted 1,000-fold with a carbon-free medium. Subsequently, 100 µL of each dilution was plated on 2216E agar plates to observe colony formation. For the coculture, colonies of S1616 (off-white) and M597 (gray-green) were distinguished from each other based on pigment production and selectively confirmed using 16S rRNA gene Sanger sequencing.

### Genome sequencing and analysis

Complete genome sequencing of M597, S1616, T6124, and W002 was performed using the PacBio and Illumina sequencing. In detail, DNA samples of these four strains of monoculture in marine broth 2216 were extracted using the TIANamp Genomic DNA Kit (Tiangen Biotech, China). The integrity of extracted DNA was examined by agarose gel electrophoresis. Subsequently, NEBNext UltraTM DNA Library Prep Kit (New England Biolabs, USA) was used to prepare the DNA library and Illumina sequencing was performed using the NovaSeq platform at the Novogene (China). The sequencing experiment generated 2 Gb sequence data for each strain. PacBio single-molecular real-time (SMRT) sequencing was conducted to construct a 10-kbp SMRT Bell library using the SMRT Bell TM Template kit. The library was sequenced following the circular consensus sequencing (CCS) strategy. This sequencing experiment resulted in a generation of 1 Gb data per strain. Preliminary genome assembly was performed by SMRT Link v5.0.1 (CCS = 3, minimum accuracy > 0.99) using the PacBio data. Subsequently, Illumina data were utilized to correct the assembled genomes using Minimap (version 2.26) (35) to obtain the complete genomes of the strains. Taxonomic classification was done by using GTDB-Tk (version 0.3.2) (36). Barrnap (version 0.9, https://github.com/tseemann/barrnap) and Aragorn (version 1.2.38) (37) were used to predict the rRNA and tRNA genes in the genomes, respectively. A closed-end ORF prediction model was employed to predict the ORFs and corresponding proteins using Prodigal (version 2.60) (38). For functional gene annotation, ORFs were employed to run a BLASTp (E-value < 1e-7) search against the KEGG database, and then the metabolic pathways were reconstructed in the KEGG Mapper (https://www.genome.jp/kegg/mapper.html). The circular genome maps were drawn using CGview (https://stothardresearch.ca/cgview/) (39).

### Phylogenetic tree construction

The 26, 2, 30, and 22 genomes belonging to *Leisingera*, *Alloyangia*, *Sulfitobacter,* and *Roseibium*, respectively, were downloaded from NCBI to construct a phylogenetic tree with M597, S1616, T6124, and W002. The 31 housekeeping genes (*dnaG, frr, infC, nusA, pgk, pyrG, rplA, rplB, rplC, rplD, rplE, rplF, rplK, rplL, rplM, rplN, rplP, rplS, rplT, rpmA, rpoB, rpsB, rpsC, rpsE, rpsI, rpsJ, rpsK, rpsM, rpsS, smpB,* and *tsf*) were extracted from all the genomes by using AMPHORA2 (40). Protein sequences were aligned individually using the software MEGA (version 7.0.26) (41) and then manually linked together. The tree was built using the maximum-likelihood method with 500 bootstrap replicates. The tree was then visualized using the software iTOL (version 6) (42).

### Sequencing and analysis of 16S rRNA gene amplicons

To determine the relative abundance of S1616 and M597 in the cocultured consortia, 16S rRNA gene amplicon sequencing was performed for the samples collected on days 0, 2, 4, 6, 8, 10, 12, and 14. DNA was extracted from the cocultures using the TIANamp Genomic DNA Kit. The V3–V4 regions of the 16S rRNA genes of extracted DNA samples were amplified using the universal primers 16S-341F (5′-CCTACGGGNGGCWGCAG-3′) and 16S-805R (5′-GACTACHVGGGTATCTAATCC-3′) and the PlatinumTM Taq DNA Polymerase (DNA free; Thermo Scientific, USA). DNA libraries were prepared using the TruSeq DNA PCR-Free Sample Preparation Kit (Illumina, USA) and sequenced on the Illumina PE250 platform in Novogene (China). More than 10,000 pair-ended reads were generated for each sample. These reads were subsequently merged using FLASH (version 1.2.11) (43). To remove the chimera sequences, merged sequences were searched against the Silva database (version 138.1) (44) using the UCHIME algorithm (version 10) (45). Classification of the sequences was carried out by mapping these sequences to the 16S rRNA genes of the S1616 and M597 using BLASTn (E-value < 1e-7 and identity > 99%). Relative abundances of the two strains in the coculture were indicated by the numbers of amplicon reads normalized to the 16S rRNA gene copy numbers in the genomes. This experiment was conducted using two biological replicates, corresponding to two amplicon data files for each time point. In total, 16 amplicon data files were obtained.

### Measurement of D-glucuronic acid consumption

The 48 h D-glucuronic acid consumption rate was measured for monocultures of S1616 and M597 and their coculture. The supernatants of the three culture groups were obtained after centrifugation at 3,687 × *g* for 10 min. These supernatants were filtered through 0.22 µm polycarbonate membrane filters (Millipore, USA) three times, and then the concentration of D-glucuronic acid in the supernatants was measured using an assay kit of D-glucuronic acid (Megazyme, Ireland). Standard samples with D-gluconic acid concentrations of 0.001, 0.005, 0.01, 0.05, 0.1, 0.5, and 1 g/L were prepared. In 96-well plates, supernatants and standard samples were mixed well with reaction buffer1 provided in the kit and allowed to react at room temperature for 5 min. Absorbance values of the supernatants and standard sample mixtures were recorded at 340 nm using a microplate spectrophotometer (Multiskan FC, Thermo Fisher Scientific, USA) as A1_supernatant_ and A1_standard_. Then reaction buffer2 was added to all the samples and allowed to react at room temperature for 5 min. The absorbance values of the mixtures were recorded again at 340 nm as A2_supernatant_ and A2_standard_. A standard curve was plotted between the absorbance (A2_standard_–A1_standard_) and D-gluconic acid concentrations of the standard samples. This standard cure was used to determine the concentrations of D-gluconic acid in the supernatants based on the variations between the A2_supernatant_ and the A1_supernatant_. The experiment was conducted on three independent biological replicates for each sample.

### Measurement of branched-chain amino acid consumption

Strain S1616 was cultured in D-glucuronic acid for 48 h, and the supernatant was extracted following the steps mentioned above. The concentration of L-leucine, L-isoleucine, and L-valine in the S1616 supernatant was measured. The S1616 supernatant was used to culture S1616 and M597 individually for an additional 48 h, followed by supernatant extraction and concentration measurement of the three branched-chain amino acids. The amino acid concentration was measured using ultra-high-performance liquid chromatography-tandem mass spectrometry (UHPLC-MS/MS) on an Agilent 1290 Infinity II UHPLC system linked to a 6470A Triple Quadrupole mass spectrometry at the Profleader Biotech Company (Shanghai, China). The detailed steps were documented in our previous study (24).

### Sequencing and analysis of transcriptomes

For transcriptomic analysis, samples of S1616 monoculture and coculture with M597 in D-gluconic acid were collected after 4 days of incubation (at early stationary phase). Bacterial cells were harvested by centrifuging the culture samples at 3,687 × *g* for 10 min, and the harvested cells were immediately frozen in liquid nitrogen. The Tiangen RNAprep Pure Cell/Bacteria Kit (China) was used to extract total RNA from the bacterial cell. The mRNA was enriched by mixing the biotin-labeled oligonucleotides complementary to rRNA with the total RNA. Subsequently, the NEBNext Ultra RNA Library Prep Kit was used to prepare RNA-Seq libraries, which were sequenced at Novogene (China) using the Novaseq 6000 system. A total of 150 bp paired-end reads were generated, and 1 Gb sequence data were obtained for each monoculture sample, while 2 Gb data were generated for each coculture sample. NGS QC Toolkit (version 2.0) (46) was used to check the quality of reads and remove the low-quality (quality score < 20) reads from the raw transcriptomics data. ORFs derived from the S1616 genome were indexed separately using Bowtie 2 (version 2.5.2) (47). Samtools (version 1.11) (48) was used to determine the number of reads mapped to each ORF. RPKM values for genes were calculated using the following formula:

RPKM = number of recruited reads/(gene length/1,000 × total number of reads/1,000,000).

Significantly differentially expressed genes of S1616 in monoculture and coculture were identified using a two-tailed Students’ *t*-test (*P*-value < 0.05 and fold change > 4). *P*-values were corrected with FDR using the “fdrtool” package in R software. A volcano plot presenting the up-regulated, down-regulated, and unchanged genes of S1616 was drawn. The top 40 (based on the fold change) KEGG-annotated differentially expressed genes were clustered using Cluster 3.0 (49), where hierarchical clustering with average linkage was used, and then visualized in Java TreeView 3.0 (50). Each culture group had three biological replicates, corresponding to three transcriptomes, thus resulting in six transcriptomes.

### Supernatant culture experiment

To examine if M597 can grow by utilizing the metabolites of S1616, the supernatant of S1616 cultured in D-gluconic acid (0.1%) for 4 days (early stationary phase) was obtained by centrifugation at 3,687 × *g* for 10 min. This supernatant was filtered through 0.22 µm polycarbonate membrane filters (Millipore, USA) three times. Simultaneously, M597 bacterial cells were washed two times using carbon-free medium. Then, the washed M597 cells were added to the supernatant of S1616. M597 culture in D-gluconic acid (0.1%) was set as the control group. The OD_600_ values of both experiment group and control were measured every 12 h. This experiment was conducted in three batches, using three independent biological replicates for each group.

### Gene mutation

Gene knockout was performed by following the method described in our previous studies (51, 52). In detail, the 877 bp upstream and the 821 bp downstream regions of the *leuC* gene of S1616 were amplified using the primer pairs *leuC*-up-F/*leuC-*up-R and *leuC*-down-F/*leuC*-down-R, respectively (Table S5). The PCR reaction mix (50 µL) consisted of 2 µL of each primer, 25 µL of super-fidelity DNA polymerase (Vazyme, China), 2 µL of DNA, and 15 µL of ddH_2_O. The upstream and downstream fragments were linked by overlap PCR in a 50 µL reaction system using the primer pair *leuC*-up-F and *leuC*-down-R. The PCR products and plasmid pDM4 were digested using the SacI and SpeI restriction enzymes at 37°C for 16 h. Subsequently, the products were linked together using T4 ligase at 16°C for 16 h to construct the knockout plasmid pDM4-*ΔleuC*. This knockout plasmid was transformed into the competent cells of *Escherichia coli* S17-1 through heat shock at 42°C for 90 s. The conjugative transfer between S17-1(pDM4-*ΔleuC*) and wild-type S1616 was performed on 2216E at 28°C for 48 h. The conjugated products were resuspended in PBS and plated on 2216E agar plates containing 100 µg/mL carbenicillin disodium (to inhibit the growth of S17-1) and 30 µg/mL chloramphenicol (to inhibit the growth of wild-type S1616). The plates were incubated at 28°C for 24 h. Successful recombinants were identified using PCR and the identified recombinants were further grown on 2216E agar plates with 15% sucrose, at 28°C for 16 h. Successful mutants were identified by PCR using *leuC*-up-F and *leuC*-down-R primers. The obtained PCR products were subjected to Sanger sequencing to confirm the mutations in the gene sequences. Another mutant strain, S1616*ΔleuD*, was obtained by following the same procedure using respective primer pairs (Table S5).

### Analysis of the marine biofilm metagenomes

The distribution patterns of S1616, M597, W002, and T6124 in 131 marine biofilms were analyzed by integrating the data obtained through the genomic and metagenomic analyses. The metagenomic data sets were generated in our previous studies (15, 18, 22). Information on the metagenomes is given in Table S4 and classified into four groups based on the type of substrates for biofilm development. All metagenomes were normalized into 1,000,000 reads per metagenome with a read length of 101 bp, and mapped to the chromosomes of S1616, M597, T6124, and W002 using BBmap (version 35.85) (53). Spearman correlation was analyzed for each pair of the strains, based on the recruited metagenomic reads numbers by the strains. The Spearman coefficient values were calculated using the “stats” package in R, and the *P*-values were corrected with FDR using the “fdrtool” package in R.

## Data Availability

The complete genome sequences of S1616, M597, W002, and T6124 were deposited in NCBI under the following accession codes: CP128601-CP128604, CP081044-CP081050, CP081126-CP081129, and CP154650-CP154655, respectively. The transcriptomic data sets (six samples) generated in this study have been deposited in CNCB under CRA013985.

## SUPPLEMENTAL MATERIAL

Supplemental figures aem.02411-24-s0001.docx Figures S1 to S10.

Supplemental tables aem.02411-24-s0002.xlsx Tables S1 to S5.

ASM does not own the copyrights to Supplemental Material that may be linked to, or accessed through, an article. The authors have granted ASM a non-exclusive, world-wide license to publish the Supplemental Material files. Please contact the corresponding author directly for reuse.
