# Supplementary material for: Branched-chain amino acid metabolism supports Roseobacteraceae positive interactions in marine biofilms
Source: Appl Environ Microbiol. 2025 Feb 11;91(3):e02411-24. doi: 10.1128/aem.02411-24 (PMC11921356; doi:10.1128/aem.02411-24)
Supplement: Supplemental figures — Figures S1 to S10. [file aem.02411-24-s0001.docx]

Fig. S1 Chromosome profiles of the four strains used for coculture experiments. The full names are: *Leisingera aquaemixtae* M597, *Roseibium aggregatum* S1616, *Alloyangia pacifica* T6124, and *Sulfitobacter indolifex* W002.

Fig. S2 Phylogenetic tree including the four strains and 80 reference strains downloaded from NCBI. The tree was built using maximum-likelihood method after alignment of 31 essential marker genes extracted from the genomes. Bootstrap values derived from 500 replicates are shown on the branches.


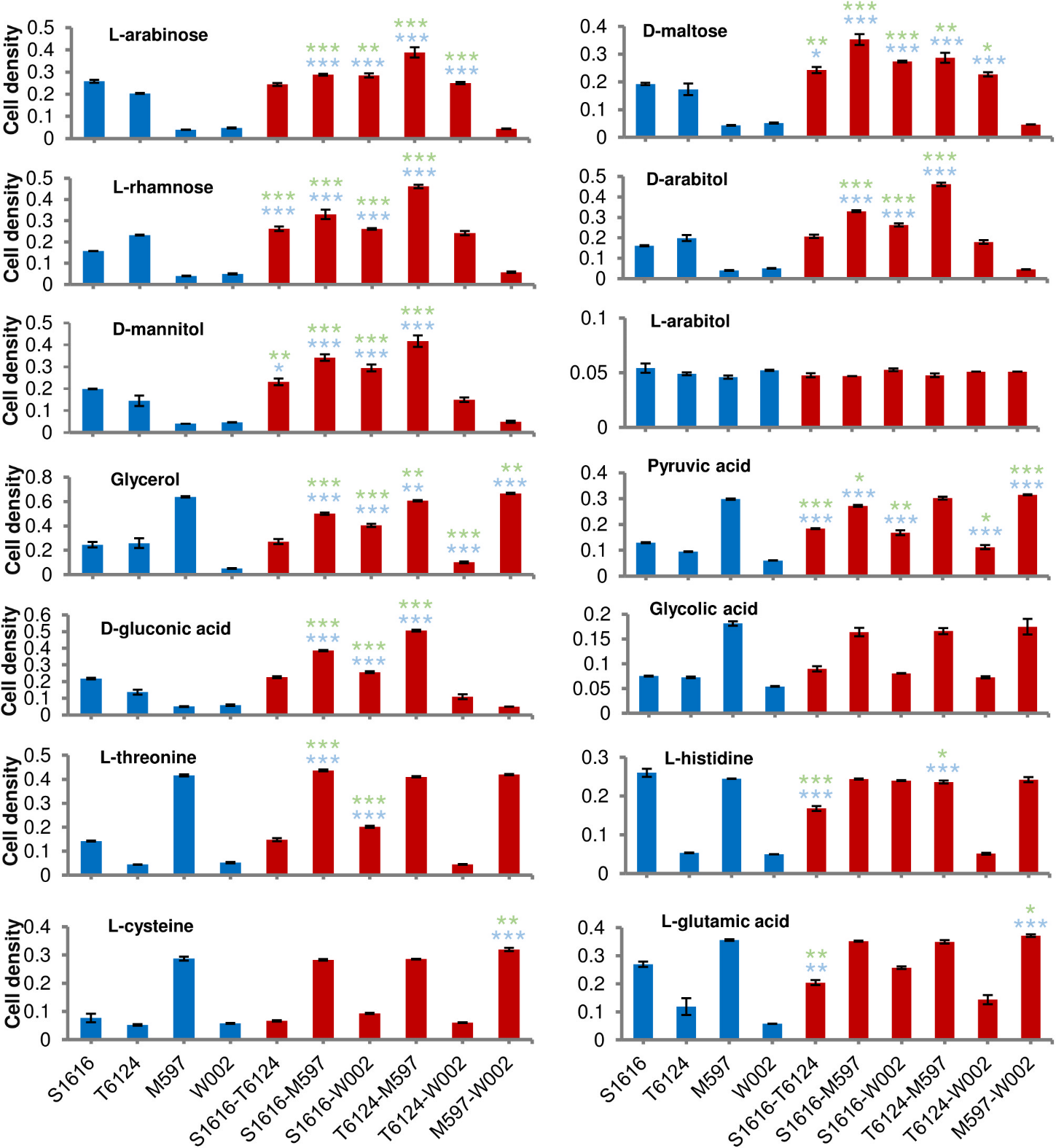


Fig. S3 Interactions among the four strains during coculture experiments with 14 sole carbon sources. The bar charts show cell densities of monocultures and cocultures. Taxonomic affiliations are *Leisingera aquaemixtae* M597, *Roseibium aggregatum* S1616, *Salipiger pacificus* T6124, and *Sulfitobacter indolifex* W002. Each given value in the bar chats is the maximum OD_600_ during the growth. The experiments were conducted with three biological replicates. Two-tailed Students’ t-test was used to analyze the difference between cell densities in monoculture and coculture. * indicates P-value < 0.05, ** indicates P-value < 0.01, and *** indicates P-value < 0.001. Stars in green and blue indicate comparison of the coculture with the two respective monocultures one by one. P-values were corrected with FDR method.

Fig. S4 Scatter plot of interaction mode statistics. The red dots represent positive interactions, the blue dots represent negative interactions, and the gray dots represent neutrality. The values corresponding to all points here are the average of the three biological replicates with the maximum OD_600_ value.


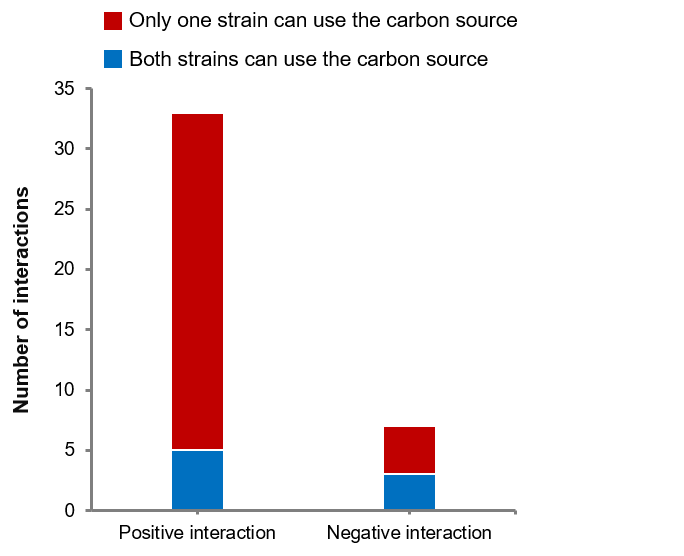


Fig. S5 In coculture, the number of positive and negative interactions corresponding to scenarios where only one strain can utilize the carbon source or both strains can utilize the same carbon source.


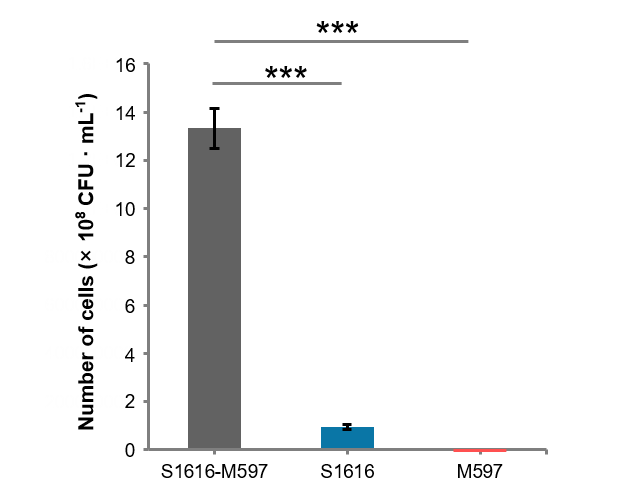


Fig. S6 The colony-forming unit (CFU) counts for S1616 and M597 during coculture and monoculture. The time points at which S1616 reached its maximum cell density (OD_600_) during monoculture (144 h) and coculture with M597 (96 h) to perform CFU counts. M597 is unable to grow on D-gluconic acid, resulting in a CFU count of 0. The data presented in this figure represent the mean of CFU counts from the cultures, with each experimental group consisting of three biological replicates. Error bars represent standard deviations of three biological replicates. *** represents P-value < 0.001 in Students’ t-test. P-values were corrected with FDR method.

Fig. S7 Time series of relative abundances of S1616 and M597 during coculture in D-gluconic acid, as revealed by 16S rRNA gene amplicon sequencing. Average values based on two biological replicates are shown. The points in the figure represent the corresponding time points.

Fig. S8 The 48-hour consumption rate of D-gluconic acid in coculture and monocultures of S1616 and M597. Error bars represent standard deviations of three biological replicates. *** represents P-value < 0.001 in Students’ t-test. P-values were corrected with FDR method.

Fig. S9 Overview of gene transcription profiles in S1616 cocultured with M597 or cultured individually. In both cultures, D-gluconic acid was the single carbon source. Statistical analysis was performed using two-sided Student’s t-test with a threshold of fold change > 4 and P-value < 0.05. The experiment was conducted with three independent biological replicates. P-values were corrected with FDR method.


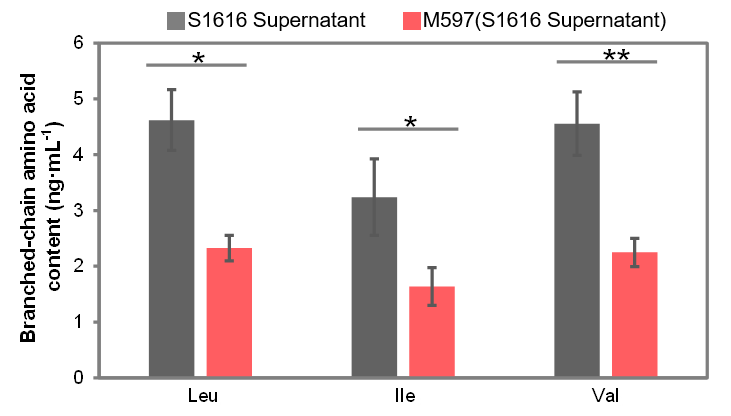


Fig. S10 Consumption of branched-chain amino acids by M597 when cultured in S1616 supernatant. The supernatant of S1616 cultured in D-gluconic acid was extracted and used to culture M597, followed by concentration measurement of L-leucine, L-isoleucine, and L-valine using metabolomics. The experiment was conducted with three biological replicates. ** and * represent P-value < 0.01 and < 0.05, respectively, in Students’ t-test. P-values were corrected with FDR method.
